# Stormy Course of Adult-Onset Still's Disease With Macrophage Activation Syndrome and Concurrent Membranoproliferative Glomerulonephritis: A Case Report

**DOI:** 10.7759/cureus.107638

**Published:** 2026-04-24

**Authors:** Abiodun W Adeyemo, Sourabh Sharma, Kudirat Busari, Opeyemi Momoh

**Affiliations:** 1 Nephrology, Zenith Medical and Kidney Centre, Abuja, NGA; 2 Nephrology, Vardhman Mahavir Medical College and Safdarjung Hospital, New Delhi, IND

**Keywords:** cytopenia, glomerulonephritis (gn), hyperferritinemia, macrophage activation syndrome (mas), still’ s disease, transaminitis

## Abstract

Adult-onset Still’s disease (AOSD) is a rare systemic inflammatory disorder of unknown etiology, characterized by fever, arthralgia, rash, and leukocytosis. Severe disease may be complicated by macrophage activation syndrome (MAS), a life-threatening hyperinflammatory state marked by cytopenias, hyperferritinemia, and multiorgan involvement. Renal manifestations in AOSD are uncommon and varied.

We report the case of a 22-year-old Nigerian male who fulfilled the Yamaguchi criteria for AOSD and presented with features consistent with MAS, including cytopenias, hyperferritinemia, hypertriglyceridemia, and transaminitis. He also developed acute kidney injury with significant proteinuria. Renal biopsy demonstrated features consistent with membranoproliferative glomerulonephritis (MPGN). The patient was successfully treated with pulsed corticosteroids followed by oral steroids and tacrolimus, resulting in clinical and biochemical improvement.

This case highlights the diagnostic complexity of AOSD complicated by MAS, particularly in the presence of atypical renal involvement. Early recognition and prompt initiation of immunosuppressive therapy are essential to improving outcomes.

## Introduction

Adult-onset Still’s disease (AOSD) is an uncommon multisystem autoinflammatory disorder of unknown etiology, first described by Bywaters in 1971 [1]. It is characterized by high-grade fever, arthralgia, an evanescent rash, leukocytosis, and negative serological markers for autoimmune disease [1,2]. The disease is rarely reported in African populations, with only a few documented cases [3-6].

The pathogenesis of AOSD involves dysregulated innate immunity and excessive cytokine release. Macrophage activation syndrome (MAS), its most severe complication, results from uncontrolled activation of macrophages and T lymphocytes, leading to a cytokine storm and multiorgan dysfunction. MAS is characterized by fever, cytopenias, hyperferritinemia, hypertriglyceridemia, and organomegaly.

Renal involvement in AOSD is uncommon but may occur, particularly in association with MAS. Reported manifestations include proteinuria, hematuria, and acute kidney injury. We present a case of AOSD complicated by MAS with concurrent membranoproliferative glomerulonephritis (MPGN), highlighting its diagnostic and therapeutic challenges.

## Case presentation

A 22-year-old Nigerian male presented with a one-month history of persistent high-grade fever, sore throat, polyarthralgia, and generalized body swelling. The joint pains involved the knees, wrists, and toes, and were associated with frothiness of urine and reduced urine output. Two weeks before presentation, he experienced generalized tonic-clonic seizures that resolved spontaneously, followed by intermittent confusion. He received empirical treatment with intravenous ceftriaxone and antimalarial medications without improvement. 

On examination, he was febrile (axillary temperature 39.20 °C) and tachycardic (pulse rate 102 bpm) and had a blood pressure of 122/68 mmHg. There was tenderness in the wrists and knees, along with bilateral pitting pedal edema. No significant lymphadenopathy was noted, and the remainder of the systemic examination was unremarkable.

The laboratory investigations revealed leukopenia(white cell count 1.2 x 10^9^/L) and thrombocytopenia (platelet count 66 x 10^9^/L). The erythrocyte sedimentation rate was elevated at 46 mm/hour, and serum ferritin was markedly high at 2,000 ng/mL (reference range: 30-400 ng/mL). Lactate dehydrogenase was elevated at 679 U/L (135-225 U/L), and triglycerides were increased at 608 mg/dL (<150 mg/dL).

Urinalysis showed 2+ protein and 2+ blood. The serum creatinine was 225 μmol/L, and the urine protein-to-creatinine ratio was 2.2 g/day. Liver function tests demonstrated transaminitis with hypoalbuminemia (Table 1).

**Table 1:** Other investigation results.

Test	Results	Reference range
Packed cell volume	42%	37%-54%
Thick-film microscopy for malaria	Negative	Negative
Stool for ova of parasite	Negative	Negative
Peripheral blood film	Polychromasia, mild rouleaux formation, leucopenia, and thrombocytopenia.	Normal
Aspartate aminotransferase	551 U/L	15-50 U/L
Alanine aminotransferase	108 U/L	5-40 U/L
Alkaline phosphatase	905 U/L	97-278 U/L
Gamma-glutamyl transferase	2,212 U/L	10-71 U/L
Total protein	3.7 g/dL	6.4-7.3 g/dL
Albumin	1.1 g/dL	3.5-5.5 g/dL
Bone marrow aspiration cytology	Negative for histiocytic hemophagocytosis	Normal

Autoimmune screening, including antinuclear antibody and rheumatoid factor, was negative. The infectious workup, including blood cultures, HIV, hepatitis B and C serology, Venereal Disease Research Laboratory (VDRL) testing, and anti-streptolysin O titer, was also negative.

**Figure 1:**
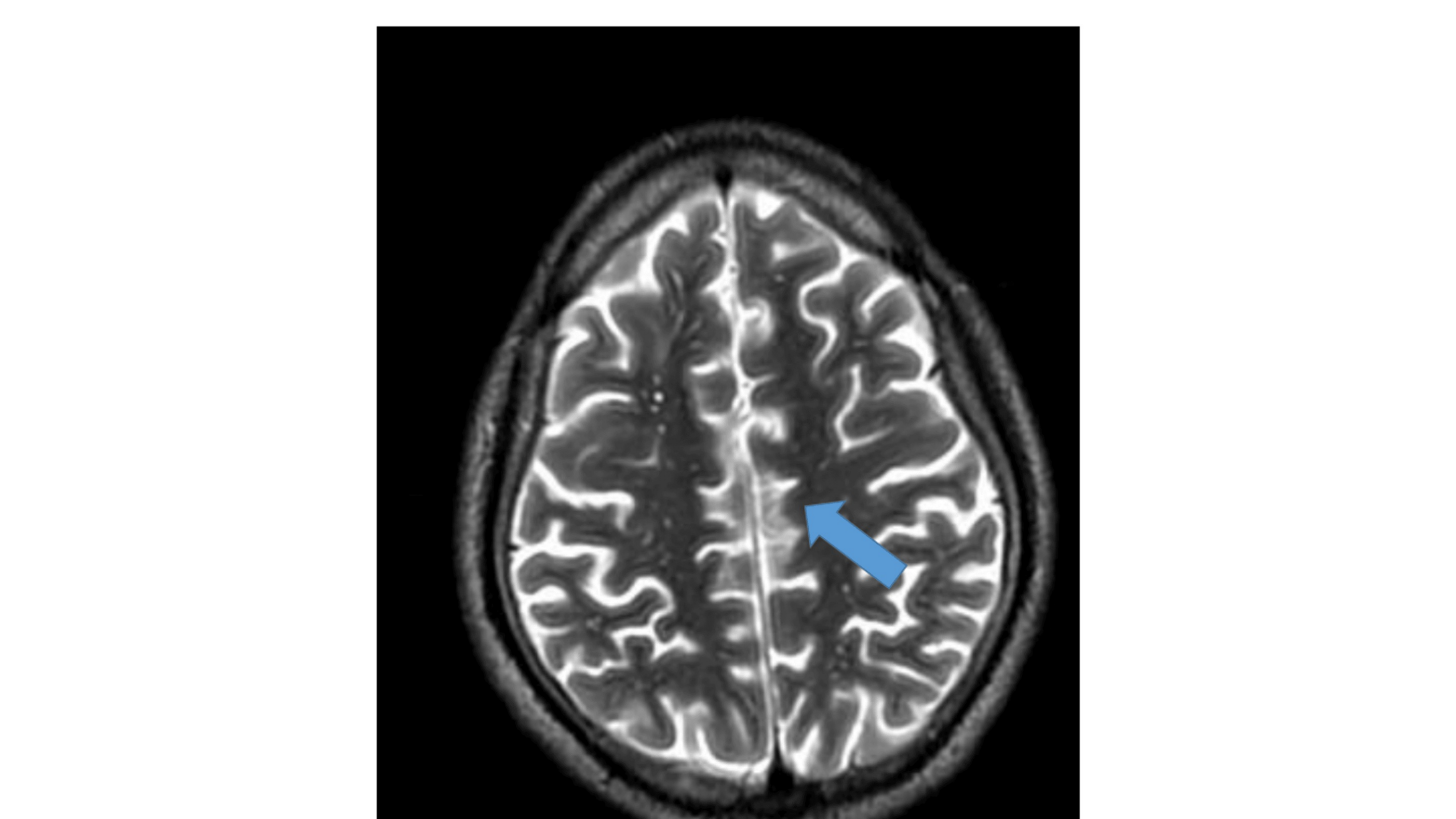
T2-weighted brain MRI of the patient showing multifocal, fairly symmetrical punctate T2 hyperintensities in the deep white matter (blue arrow).

The abdominal ultrasound scan detected mild splenomegaly and increased echoes in the liver and kidney, suggestive of an inflammatory state, while other abdominal organs appeared normal. The brain MRI showed multifocal, fairly symmetrical punctuate T2 hyperintensities in the deep white matter, indicative of encephalitis (Figure 1).

We diagnosed AOSD with concurrent MAS based on the combination of persistent high-grade fever, sore throat, arthritis, splenomegaly, transaminitis, cytopenias, hyperferritinemia, hypertriglyceridemia, elevated lactate dehydrogenase (LDH) levels, and negative autoimmune and infectious screening. Although leukopenia is atypical for AOSD, it was attributed to the development of MAS, which can suppress the usual leucocytosis seen in AOSD.

**Figure 2:**
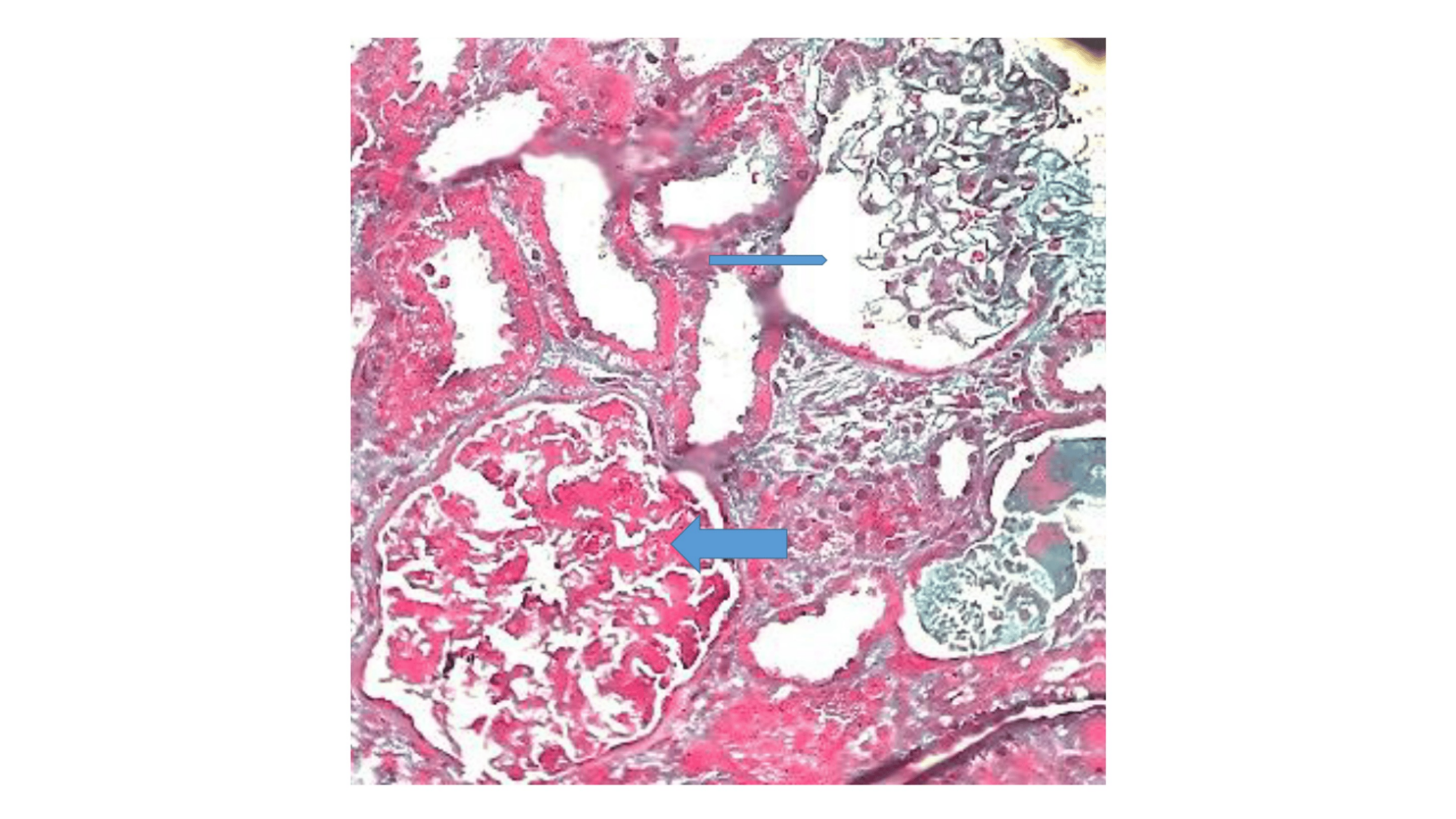
Light microscopy of renal biopsy tissue showing two glomeruli: one normal (thin arrow) and the other demonstrating mesangial proliferation (thick arrow).py of renal biopsy tissue Masson’s trichrome stain, ×200.

**Figure 3:**
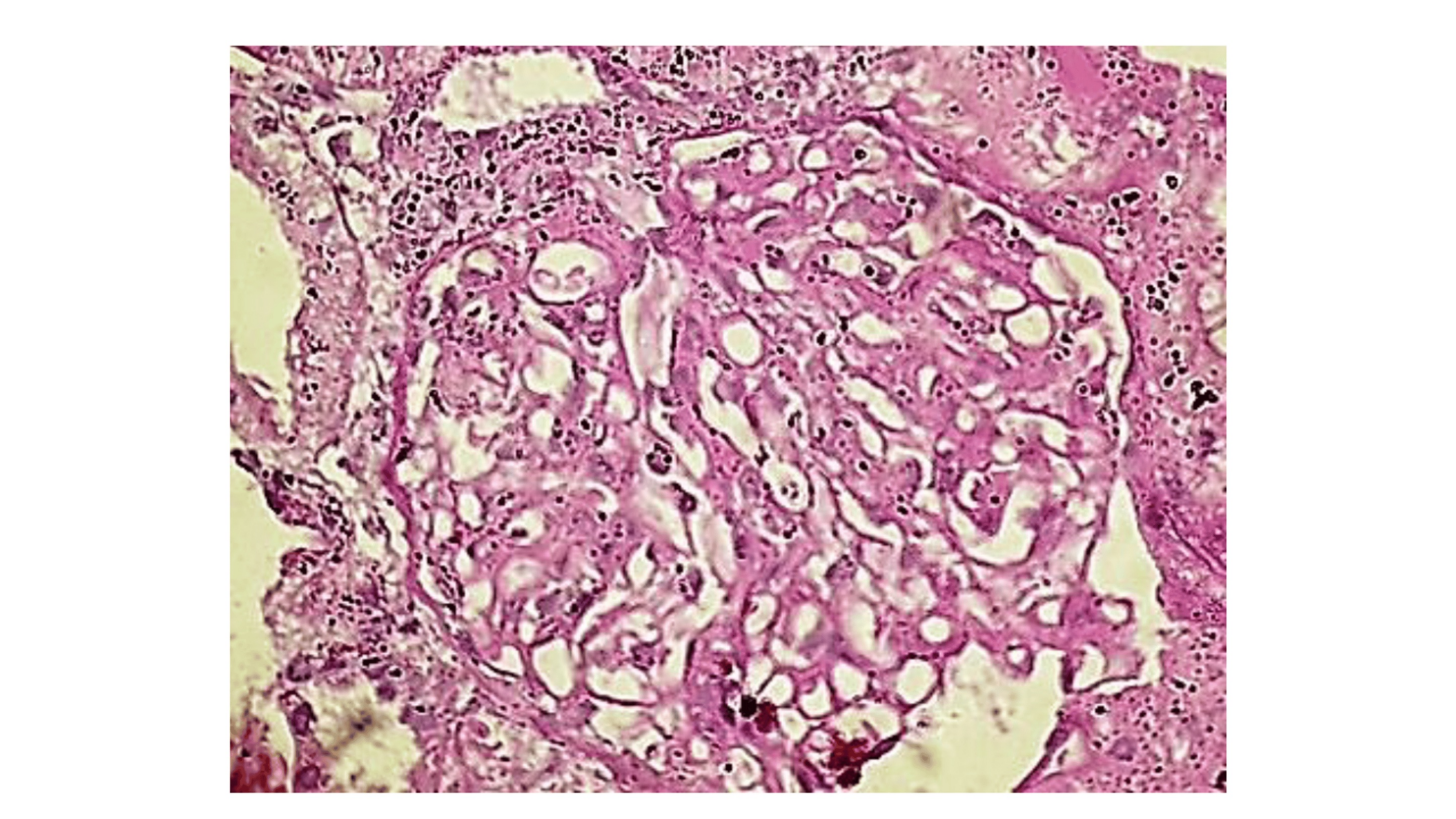
A glomerulus exhibiting mesangial and endocapillary hypercellularity with thickening of the peripheral basement membrane. Hematoxylin and eosin stain, ×400.

**Figure 4:**
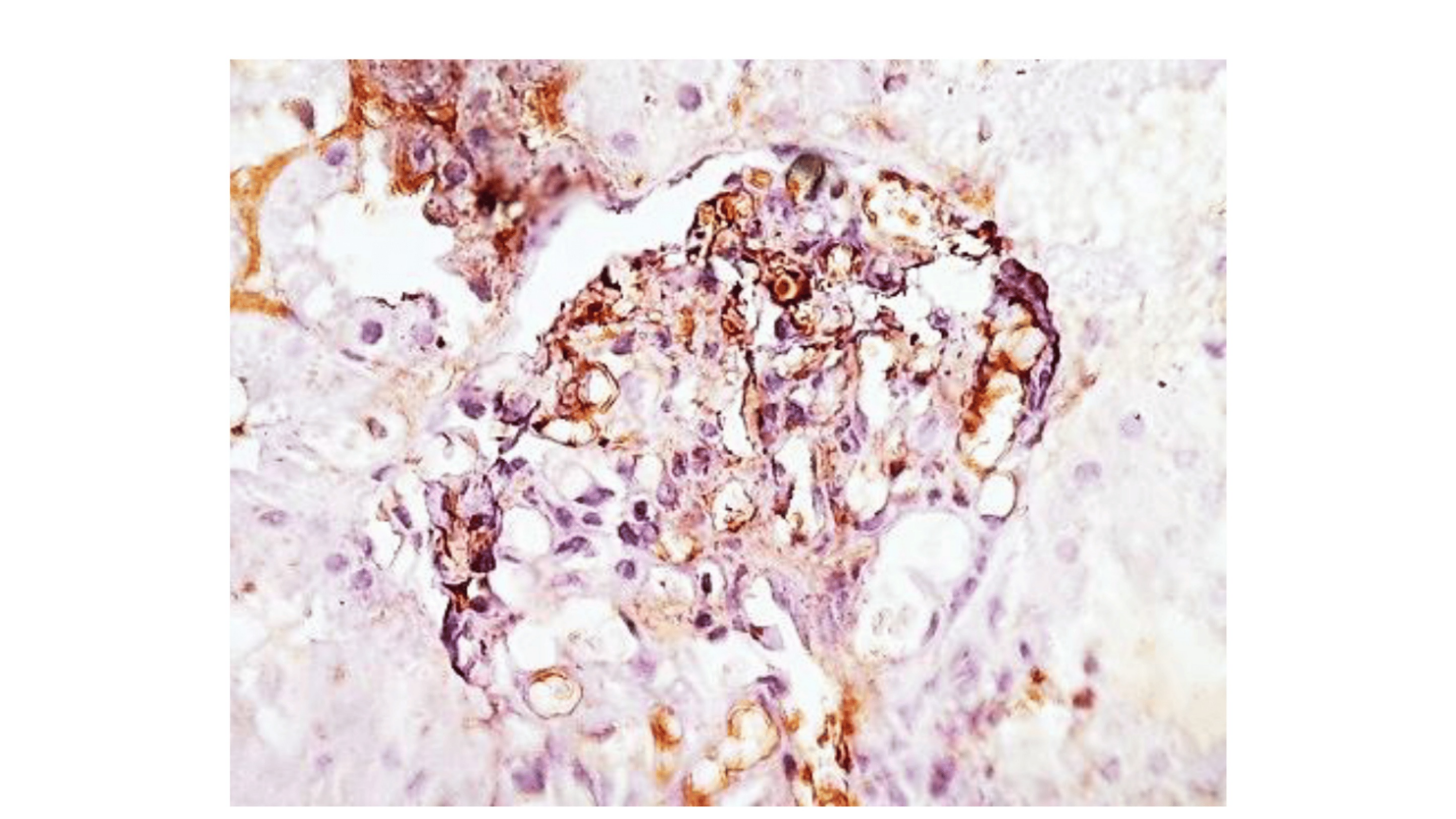
Glomerulus showing faint mesangial immune complex deposits. Anti-IgG stain, ×200.

Renal biopsy performed after resolution of thrombocytopenia revealed mesangial and endocapillary proliferation with thickening of the glomerular basement membrane, consistent with a membranoproliferative pattern of injury. Focal red blood cell casts were seen in the tubules, and immunohistochemistry demonstrated weak IgG staining with minimal immune complex deposition (Figures 2-4). The limited immune staining may reflect prior initiation of immunosuppressive therapy before biopsy.

The patient was treated with intravenous methylprednisolone (1 g daily for 3 days), followed by oral prednisolone (40 mg/day). Tacrolimus was initiated at 5 mg twice daily, achieving a trough level of 4.2 ng/mL. Telmisartan and dapaglifozin were commenced for proteinuria management. Albumin infusions and furosemide were administered for volume control.

## Discussion

AOSD is commonly diagnosed using the Yamaguchi criteria, which require at least two of the following major criteria: fever ≥39 °C for at least one week, arthralgia lasting two weeks or longer, an evanescent rash associated with febrile episodes, and leukocytosis (≥10,000/mm³ with ≥80% neutrophils); and at least three minor criteria, including pharyngitis, lymphadenopathy and/or splenomegaly, transaminitis, and negative rheumatoid factor and antinuclear antibody, in the absence of infection, malignancy, or other rheumatologic diseases [7]. Our patient fulfilled two major and four minor diagnostic criteria for AOSD. In addition, he had leukopenia and thrombocytopenia, raising suspicion for the development of MAS. Based on the 2016 European Alliance of Associations for Rheumatology criteria, the presence of fever, bicytopenia, hyperferritinemia, hypertriglyceridemia, and transaminitis fulfills the diagnostic criteria for MAS [8]. Although bone marrow examination is a useful confirmatory test that also helps exclude concomitant infections or malignancy, bone marrow aspirate microscopy was negative in our patient. While histiocytic hemophagocytosis is considered the diagnostic hallmark of MAS, it may be absent in some cases [9].

Stéphan et al. in 1993 coined the term “macrophage activation syndrome” as a variant of hemophagocytic syndrome occurring in AOSD and other inflammatory conditions [10]. MAS can lead to multiorgan dysfunction due to a cytokine storm resulting from amplified macrophage proliferation via specific toll-like receptors that activate inflammasomes (NACHT, LRR, and PYD domain-containing proteins). This process leads to the production of caspases and pro-inflammatory cytokines, including interleukins (IL-1β, IL-6, IL-8, IL-17, IL-18) and tumor necrosis factor (TNF) [11].

Renal manifestations may occur in up to a quarter of patients with AOSD, with or without concurrent MAS. When present, they may manifest as proteinuria, hematuria, and acute kidney injury, as seen in our patient [11]. In a recent systematic review of 36 patients with AOSD who underwent kidney biopsy, secondary amyloidosis (25%) was the most common finding, followed by collapsing glomerulopathy (11.4%), thrombotic microangiopathy (11.4%), IgA nephropathy (9.1%), minimal change disease (6.8%), and others [12]. Recent literature suggests that while the spectrum of renal pathology in AOSD is expanding, proliferative glomerular lesions remain uncommon and heterogeneous, with only isolated reports describing immune-mediated patterns of injury [13]. Typical pathological findings in the presence of MAS include interstitial nephritis, mesangial glomerulonephritis, collapsing glomerulopathy, and amyloidosis [14]. In our patient, the kidney biopsy demonstrated mesangial and endocapillary hypercellularity with thickened capillary walls and IgG deposition, which is consistent with an immune complex-associated MPGN. The presence of immunoglobulin deposition, even at low intensity, in the absence of C3-dominant staining, argues against complement-mediated disease and supports an immune-complex-associated mechanism [15]. The relatively low intensity of immunoglobulin staining can be understood in the context of the underlying disease, and the histological features reflect both disease activity and the modifying effect of treatment. AOSD complicated by MAS is associated with a marked cytokine-driven inflammatory response, which may result in transient or low-level immune complex formation with downstream complement activation, leading to glomerular injury despite limited detectable immunoglobulin on biopsy. In addition, prior use of immunosuppressive therapy may reduce circulating immune complexes and alter their deposition within the glomerulus, contributing to the attenuated immunohistochemistry findings [16]. The coexistence of MAS and biopsy-proven MPGN in AOSD is rarely reported, and none to our knowledge demonstrating an MPGN pattern in this clinical context has been reported in an African, thereby highlighting the uniqueness of this case. Current understanding recognizes MPGN as a pattern of glomerular injury that requires further classification based on immunopathologic findings and underlying mechanism [15]. Immune complex-mediated MPGN is characterized by deposition of immunoglobulin with complement and is most often associated with systemic inflammatory or autoimmune conditions [15].

**Figure 5:**
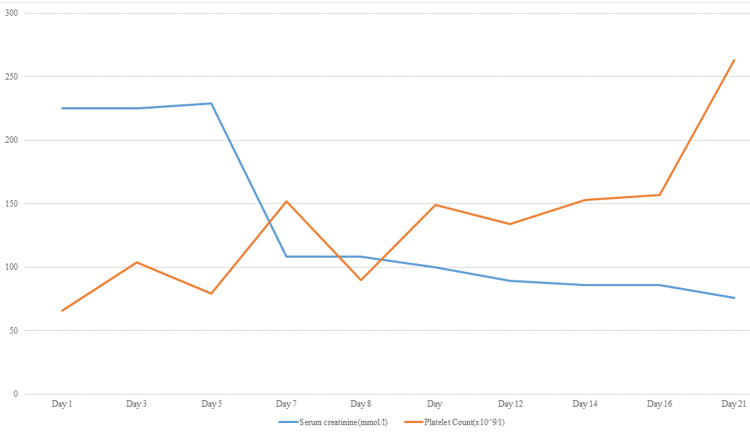
Pattern of serum creatinine and platelet count during treatment.

**Figure 6:**
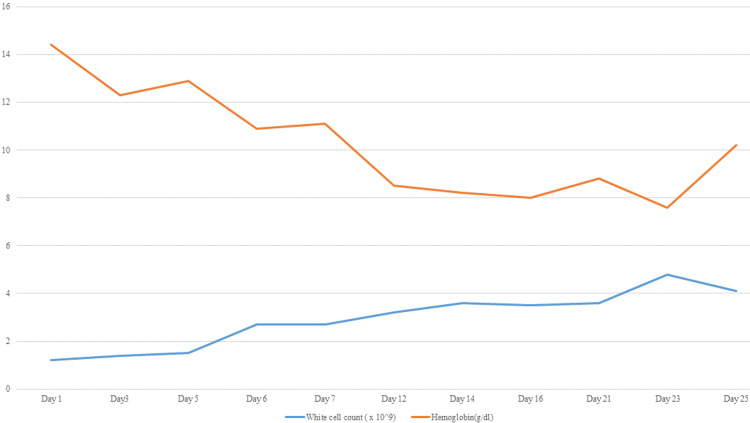
Trends in hemoglobin and white blood cell count during treatment.

The severity of MAS necessitates immediate treatment with glucocorticoids combined with a disease-modifying anti-rheumatic drug (DMARD), justifying the initiation of methylprednisolone for rapid symptom control. Although less commonly used, tacrolimus is a therapeutic option for MAS, and we selected it due to the concurrent MPGN [17,18]. Several case reports have also supported the use of cyclosporine in MAS [19,20]. At reassessment in the third week of therapy, the serum creatinine and platelet counts have significantly improved (Figure 5). Conversely, the hemoglobin level reached its nadir during the same period, necessitating the initiation of erythropoietin therapy, after which it improved as renal function normalized. Figure 6 depicts the pattern of hemoglobin and white cell count with treatment.

The patient experienced a relapse of confusion; however, a repeat brain MRI showed no new abnormalities. Oral prednisolone was substituted with dexamethasone 4 mg twice daily to enhance central nervous system penetration. The serum ferritin level remained elevated at 1,500 ng/mL. Oral dexamethasone was continued for one month and gradually tapered to 2 mg daily. During subsequent follow-up, kidney and liver function parameters remained stable, with no further episodes of confusion, fever, or arthralgia. The urine protein-creatinine ratio and serum ferritin levels improved to 0.2 g/g and 400 ng/mL, respectively.

This case underscores the importance of early recognition of MAS and proactive evaluation for renal involvement in patients with AOSD, as timely immunosuppressive therapy may prevent irreversible organ damage and improve overall outcomes.

## Conclusions

AOSD complicated by macrophage activation syndrome with concurrent renal involvement is a rare but potentially life-threatening condition. This case highlights the diagnostic complexity of Still’s disease in the presence of cytopenias, hyperferritinemia, and multiorgan involvement, particularly when renal manifestations such as membranoproliferative glomerulonephritis occur. Early recognition and prompt initiation of immunosuppressive therapy are critical to improving outcomes. Our report also underscores the potential role of calcineurin inhibitors as effective adjuncts in managing both MAS and associated glomerular disease, especially in resource-limited settings where biologic therapies may not be readily available. Clinicians should remain vigilant for renal complications in Still’s disease, as timely intervention can result in favorable clinical and renal recovery.

## References

[1] Simeni Njonnou SR, Kemta Lekpa F, Essoka Essoka AR (2025). Challenges on diagnosis and treatment of refractory adult-onset still disease in Sub-Saharan Africa: a case report. J Med Case Rep.

[2] Diogo M, Soares J, Pimentel T, Ferreira A (2010). Adult-onset Still disease as the cause of fever of unknown origin. Acta Med Port.

[3] Asanuma YF, Mimura T, Tsuboi H, Noma H, Miyoshi F, Yamamoto K, Sumida T (2015). Nationwide epidemiological survey of 169 patients with adult Still's disease in Japan. Mod Rheumatol.

[4] Akintayo RO, Adelowo O (2015). Adult-onset Still's disease in a Nigerian woman. BMJ Case Rep.

[5] Diallo S, Ka MM, Pouye A (2007). Still disease in adult: a Senegalese case report. Dakar Med.

[6] Cheikhrouhou Abdelmoula L, Tekaya R, Ben Hadj Yahia C, Chaabouni L, Zouari R (2007). Adult onset Still's disease: about 11 cases. Tunis Med.

[7] Macovei LA, Burlui A, Bratoiu I (2022). Adult-onset Still's disease-a complex disease, a challenging treatment. Int J Mol Sci.

[8] Ravelli A, Minoia F, Davì S (2016). 2016 classification criteria for macrophage activation syndrome complicating systemic juvenile idiopathic arthritis: a European League Against Rheumatism/American College of Rheumatology/Paediatric Rheumatology International Trials Organisation Collaborative Initiative. Ann Rheum Dis.

[9] Gars E, Purington N, Scott G, Chisholm K, Gratzinger D, Martin BA, Ohgami RS (2018). Bone marrow histomorphological criteria can accurately diagnose hemophagocytic lymphohistiocytosis. Haematologica.

[10] Stéphan JL, Zeller J, Hubert P, Herbelin C, Dayer JM, Prieur AM (1993). Macrophage activation syndrome and rheumatic disease in childhood: a report of four new cases. Clin Exp Rheumatol.

[11] Carter SJ, Tattersall RS, Ramanan AV (2019). Macrophage activation syndrome in adults: recent advances in pathophysiology, diagnosis and treatment. Rheumatology (Oxford).

[12] Arya PV, Marnet E, Rondla M, Tan JW, Unnikrishnan D, Buller G (2024). Renal manifestations in adult-onset Still's disease: a systematic review. Rheumatol Int.

[13] Gerfaud-Valentin M, Jamilloux Y, Iwaz J, Sève P (2014). Adult-onset Still's disease. Autoimmun Rev.

[14] Lisa AM (2025). Still’s disease in adults: Clinical Manifestations and diagnosis. https://www.uptodate.com/contents/stills-disease-in-adults-clinical-manifestations-and-diagnosis?utm_medium=email&utm_source=transaction.

[15] Kidney Disease: Improving Global Outcomes (KDIGO) Glomerular Diseases Work Group (2021). KDIGO 2021 clinical practice guideline for the management of glomerular diseases. Kidney Int.

[16] Yu SM, Deoliveira M, Chung M, Lafayette R (2024). Membranoproliferative glomerulonephritis pattern of injury. Adv Kidney Dis Health.

[17] Lisa AM, Peter AN (2025). Still’s disease in adults: Treatment. James RO, Siobhan MC.

[18] Shakoory B, Geerlinks A, Wilejto M (2023). The 2022 EULAR/ACR points to consider at the early stages of diagnosis and management of suspected haemophagocytic lymphohistiocytosis/macrophage activation syndrome (HLH/MAS). Ann Rheum Dis.

[19] Mouy R, Stephan JL, Pillet P, Haddad E, Hubert P, Prieur AM (1996). Efficacy of cyclosporine A in the treatment of macrophage activation syndrome in juvenile arthritis: report of five cases. J Pediatr.

[20] Ravelli A, De Benedetti F, Viola S, Martini A (1996). Macrophage activation syndrome in systemic juvenile rheumatoid arthritis successfully treated with cyclosporine. J Pediatr.

